# Designing Functional Fruit-Based Recovery Drinks in Powder Form That Contain Electrolytes, Peptides, Carbohydrates and Prebiotic Fiber Taking into Account Each Component’s Osmolality

**DOI:** 10.3390/molecules26185607

**Published:** 2021-09-15

**Authors:** Anna Sadowska, Franciszek Świderski, Klaudia Kulik, Bożena Waszkiewicz-Robak

**Affiliations:** Department of Functional and Organic Food, Institute of Human Nutrition Sciences, Warsaw University of Life Sciences, Nowoursynowska Str. 159c, 02-776 Warsaw, Poland; franciszek_swiderski@sggw.edu.pl (F.Ś.); klaudia_kulik@sggw.edu.pl (K.K.)

**Keywords:** osmolality, raspberries powders, recovery drinks

## Abstract

High levels of osmolalities have been found in manufactured carbohydrate-based functional drinks that occasionally include added protein; however, fruit components rich in bioactive ingredients have been absent. It has proved difficult to obtain recovery drinks based on natural fruit components that deliver calories and nutrients to the body whilst simultaneously ensuring that the body is adequately hydrated after physical exertion; the problem being that it is difficult to ensure the drinks’ stability at low pH levels and maintain an appropriate sensory quality. This study aims to develop drinks based on natural fruit components that contain added electrolytes, carbohydrates, prebiotic fiber and protein; an improved water and electrolyte balance; the calories needed after intense physical exertion; a high content of nutrients; and a favorable sensory quality. Furthermore, the relationships between regressive osmolalities of beverage components are herein investigated. The study materials were raspberry powders (prepared via fluidized-bed jet milling, drying, freeze-drying and spray-drying) as well as citrated sodium, potassium, magnesium salts, isomaltulose, hydrolyzed collagen, whey protein isolate and prebiotic fiber. The drinks’ polyphenols and antioxidant properties were measured spectrophotometrically, whilst vitamin C content was determined using high-pressure liquid chromatography. The sensory qualities of each drink were assessed according to a scaling method. Six test versions of recovery drinks were prepared in which osmolalities ranged from 388 to 607 mOsm/kg water, total polyphenol content was 27–49 mg GAE/100 mL and vitamin C level was 8.1–20.6 mg/100 mL, following compositions defined by the study results. It is thus possible to obtain fruit-based recovery drinks of the recommended osmolality that contain added protein, prebiotics and fiber, as well as defined amounts of electrolytes and carbohydrates. All drinks were found to have a satisfactorily sensory quality. The design of appropriate recovery drink compositions was also greatly helped by investigating the relationships among the regressive osmolalities of beverage components (i.e., electrolytes, carbohydrates, fruit powders and protein).

## 1. Introduction

After finishing any physical exertion, due care and attention should be taken to ensure the regeneration of the body’s tissues and replenishing of used-up energy reserves, without any excess intake of calories. A good idea is to take regenerating sports drinks containing added protein, which have osmolalities that are considered adequate for hydrating the body [1]. Accounting for osmolality is especially important whenever the body becomes dehydrated or faces other challenging events [2]. An important part of retailed functional beverages are sports drinks with osmolalities ranging from 275 to 295 mOsm/kg water and marketed for their abilities to replenish electrolytes lost during exercise, deliver carbohydrates, prevent dehydration and sustain endurance capacity [3,4,5,6]. As the name implies, isotonic drinks have similar osmolalities to bodily fluids, whereas ‘hypotonic’ ones possess osmolalities <275 mOsm/kg water, which include various ‘light’ sugar-free drinks/mineral waters. The latter are quite obviously unable to rapidly correct water–electrolyte balance because of their inherently lowered electrolyte concentrations, as opposed to isotonic drinks. It is recommended that persons engaged in moderate physical exercise/work should drink hypotonic beverages when not inordinately sweating. Guidelines for oral rehydration currently include drinking hypotonic drinks if rapid replacement of fluid is required [7,8,9]. However, if fluid balance needs to be maintained over a relatively longer time, such drinks may not be appropriate because of the varying urine excretion. A key role in fluid homeostasis is played by electrolytes, particularly sodium, where the excretory losses in sodium and chlorine are higher compared to those of magnesium, calcium and potassium. Hyponatremia can arise from excessive irrigation with clean water [10,11,12,13], but not hypokalemia, nor hypomagnesaemia. Sports drinks contain carbohydrates and mainly sodium and potassium ions as electrolytes [14], with sodium being the ion that chiefly determines serum osmolality. Increased sodium concentration stimulates thirst [15]. Urdampilletai et al. (2015) [16] reported that in order to ensure an optimal content of the Na^+^ ion in the human body, the sodium content in drinks should be 0.5–1.5 g/L, while isotonic drinks should be 0.5–0.7 g/L and hypertonic drinks should be 1.0–1.5 g/L. It thus follows that soft drinks or fruit juices that contain high amounts of carbohydrates are unsuitable as functional rehydration drinks; indeed, the majority of fruit juices and soft drinks have levels >100 g carbohydrates/L and are also hypertonic, as are energy drinks with osmolalities ranging 500–800 mOsm/kg. Carbohydrate concentrations of 60–80 g/L have been recommended in rehydration drinks by studies on performance and fatigue to deliver optimal support [10,17,18,19]. Hypertonic drinks with high osmolality pressure are not recommended for athletes because water absorption rates become decreased, which in turn leads to a risk of gastrointestinal discomfort. Despite this, these drinks may be consumed in restricted amounts for the renewal of glycogen stores [20,21]. Evans et al. (2009) [7] found that a hypertonic 10% glucose-electrolyte solution of around 667 mOsm/kg proved more effective at maintaining rehydration following exercise-induced dehydration for a body mass around 1.9% than a 2% glucose solution of an osmolality that was approximately 193 mOsm/kg, or a 0% glucose solution with an osmolality of around 79 mOsm/kg.

Literature data are very scant on how the composition of recovery drinks is designed and whether the osmolalities of individual carbohydrate and protein drink components can be used to predict overall drink osmolalities. Studies have only recently measured the effect of carbohydrate osmolalities from various beverages on physiological body/animal hydration [3,15,16,22]. There has also been no data published on the ability of a fruit powder that has been dissolved in water to aid a body’s recovery, as the powder provides fruit-derived carbohydrates, whilst also containing electrolytes and additional protein or fiber components. The presented study continues on from our previous research [22] concerning whether powdered black currant fruits could be used to design body-regenerating beverages. That study did not include the measurement of osmolalities, nor did it determine whether any added substances could be used to supplement beverage sweetness, including protein, isomaltulose (with a reduced glycemic index (GI)) and soluble fiber (e.g., inulin with prebiotic properties), or certain electrolytes such as citrates of sodium, potassium and magnesium. Our previous study did not measure osmolalities in powders derived from dried raspberries using the fluidized-bed jet milling and drying (FBJD), freeze-drying (FD) and spray-drying (SD) methods. The present study focuses on whether it is possible to design a fruit-based and nutrient-rich regenerating drink (e.g., with protein and fiber) that contains the bioactive properties of so-called ‘super fruit’ derived powders and possesses a suitable osmolality for ensuring adequate hydration of the body after intensive exercise. This study aims to design a functional recovery drink based on natural fruit components that contains added electrolytes, carbohydrates, prebiotic fiber and protein—so as to improve water and electrolyte balance and provide the required calories after intense physical exertion—while also ensuring a high nutrient content and delivering a good sensory quality. Relationships were also investigated between regressive osmolalities of beverage components (such as electrolytes, carbohydrates, fruit powders and protein) to determine whether they could be used to design a composition suitable for recovery beverages.

## 2. Results and Discussion

### 2.1. Osmolality of Recovery Drink Components

**Figure 1:**
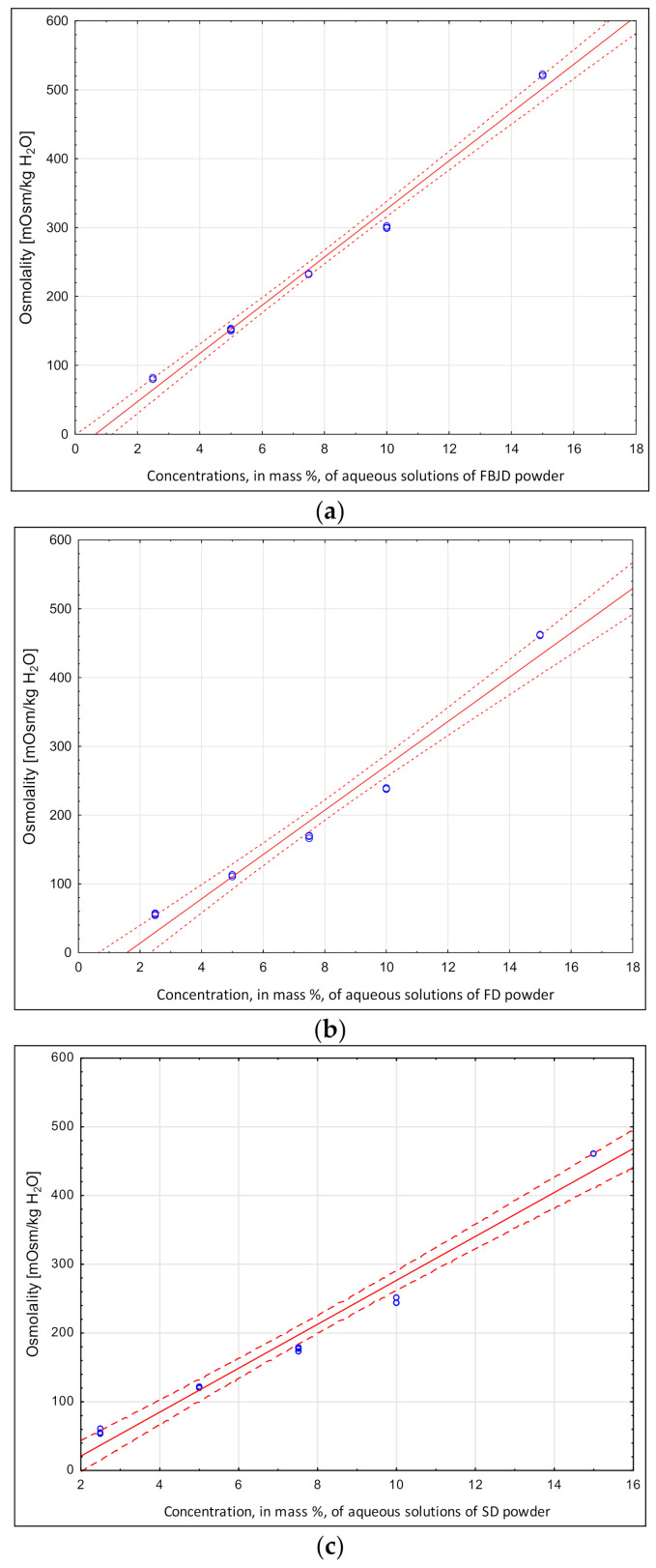
Raspberry powder osmolalities in aqueous solution. (**a**) FBJD powder: Osmolality = −22.53 + 34.96 × Concentration (%); r^2^ = 0.993. (**b**) FD powder: Osmolality = −50.55 + 32.23 × Concentration (%); r^2^ = 0.984. (**c**) SD powder: Osmolality = −43.05 + 31.96 × Concentration (%); r^2^ = 0.987.

There is a need to design recovery beverages with fruit components, proteins and minerals appropriate for an osmolality that will produce a desired pH. This, however, limits the amounts of high-acidity fruits that can be employed. Obtaining the desired osmolality for this type of beverage requires selecting and determining the proper concentrations of added minerals (electrolytes) and carbohydrate components necessary to ensure the correct sweetness. When proteins are introduced, acidity needs to be kept below their isoelectric points. This study measured the osmolality of components selected from the abovementioned groups according to which they were added as ingredients to different functional drinks. Fruit powders were either obtained from whole fruits using the new FBJD and well-known FD methods, or from raspberry juice using the SD method, containing maltodextrin as a carrier. Figure 1a–c show the osmolalities of powders obtained using abovementioned methods, where similar values were observed irrespective of method despite the large technological differences in obtaining these powders. The fruits were freeze-dried then ground to the same granulation using both the FD method and FBJD method. In the latter, the powders are obtained in a strong air stream, causing simultaneous drying and grinding [23]. SD powders were obtained by spraying a liquid product onto the carrier in a drying chamber through which a hot drying agent flowed, causing the solvent to rapidly evaporate from the droplets and thereby creating particles of powder that dropped to the bottom of the chamber [22]. Slightly higher osmolalities were measured for the FBJD-obtained powders than for to powders acquired using the FD and SD methods. This may have been due to differences in the microstructures of the powders, as well as their higher solubility when compared with the FD powders [23]. The osmolality of powders is mainly influenced by their content of water-soluble particles, especially sugars, followed by their acidic content [22]. Similar relationships between the osmolality of FD- and SD-obtained powders were found in previous studies Sadowska et al. (2020) [22] for powders derived from black currants. Our osmolality results show that the fruit powders could be added in significant amounts when designing functional drinks with controlled osmolality, and that they allowed the addition of other necessary components, especially electrolytes and carbohydrates, which provide calories and are essential to ensuring the desired sensory quality of a beverage.

**Figure 2:**
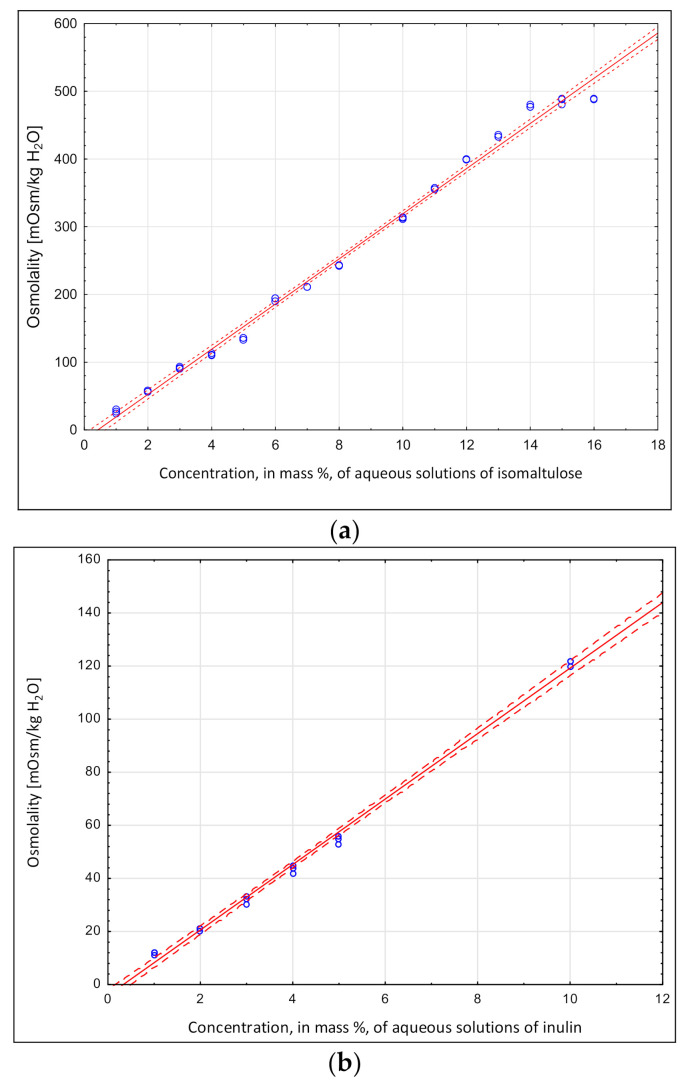
Isomaltulose. (**a**) Osmolality = −14.19 + 33.35 × Concentration; r^2^ = 0.996 and inulin. (**b**) Osmolalities in aqueous solution: Osmolality = −14.19 + 33.35 × Concentration; r^2^ = 0.996.

Figure 2a,b shows the osmolalities of isomaltulose and inulin, components that are classified as being water-soluble in aqueous solutions at various concentrations’.

The functional features of these two components were taken into account when designing the beverage: isomaltulose for its reduced glycemic index compared to other simple sugars, and inulin for its prebiotic properties and desired sensory characteristics, especially its good solubility in water and sweet taste [24,25]. Linear relationships were observed between osmolality and concentration for all of the tested components, ranging from 1% to 15% for isomaltulose, and from 1% to 10% for inulin; all correlations were significant (*p* < 0.05), and the obtained relationships between the studied parameters were almost complete. Similar dependencies between osmolality on concentrations of carbohydrates such as glucose, sucrose and maltodextrin (DE23–32) had been obtained in our previous research [22]. The beverage components from this study (Figure 2a,b), however, had significantly different osmolalities. Higher values were measured for isomaltulose, which were comparable to the sucrose osmolality obtained in our aforementioned study [22]. The osmolality of isomaltulose was three to four times higher than that of the tested inulin. The carbohydrates and inulin tested indicate that they can be used to design functional drinks with defined osmolalities, including drinks for which high osmolality is undesirable such as beverages with complex compositions of fruit components and electrolytes.

**Figure 3:**
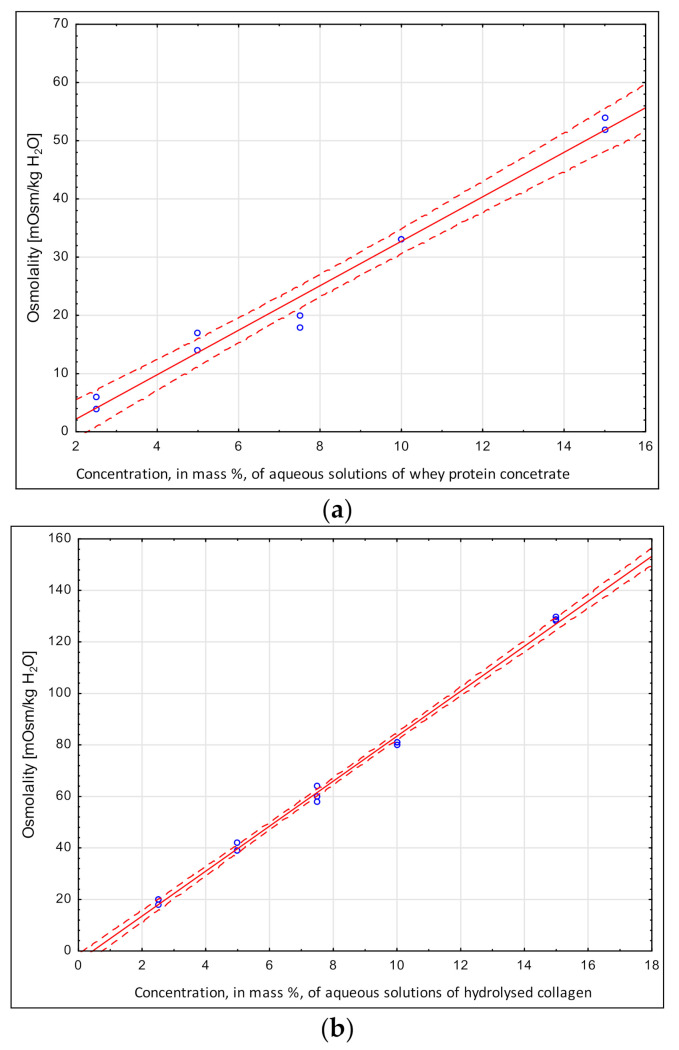
Whey protein concentrate. (**a**) Osmolality = −5.47 + 3.82 × Concentration; r^2^ = 0.989 and hydrolyzed collagen. (**b**) Osmolalities in aqueous solution: Osmolality = −5.47 + 3.82 × Concentration; r^2^ = 0.989.

Figure 3a,b shows the osmolality resulting from high concentrations of two groups of selected protein preparations: whey protein concentrate and enzymatically hydrolyzed collagen proteins. Consistent with previous studies, a significantly (*p* < 0.05) positive relationship was observed between osmolalities and the preparations’ contents in aqueous solutions, ranging from 1% to 10% added content. The osmolality of the studied proteins varied depending on their type and content. Whey proteins had lower osmolalities to that of enzymatically hydrolyzed collagen proteins. Such differences may be ascribed to the presence of proteins with different structures and sizes in these preparations. It is thereby possible to use protein preparations as an ingredient of fruit-based functional regenerating drinks, although collagen hydrolysate may be more useful from an osmolality perspective. The lower osmolality of collagen hydrolysate allows it to be used in complex systems containing electrolytes, fruit powders and carbohydrates. Another advantage is that there is no problem of protein precipitating out at the protein’s isoelectric point with the collagen preparation, which contains partially hydrolyzed proteins and thus permits larger amounts of fruit powder to be added. There are no results of studies in the available literature discussing the osmolality of protein preparations as a component of recovery drinks.

**Figure 4:**
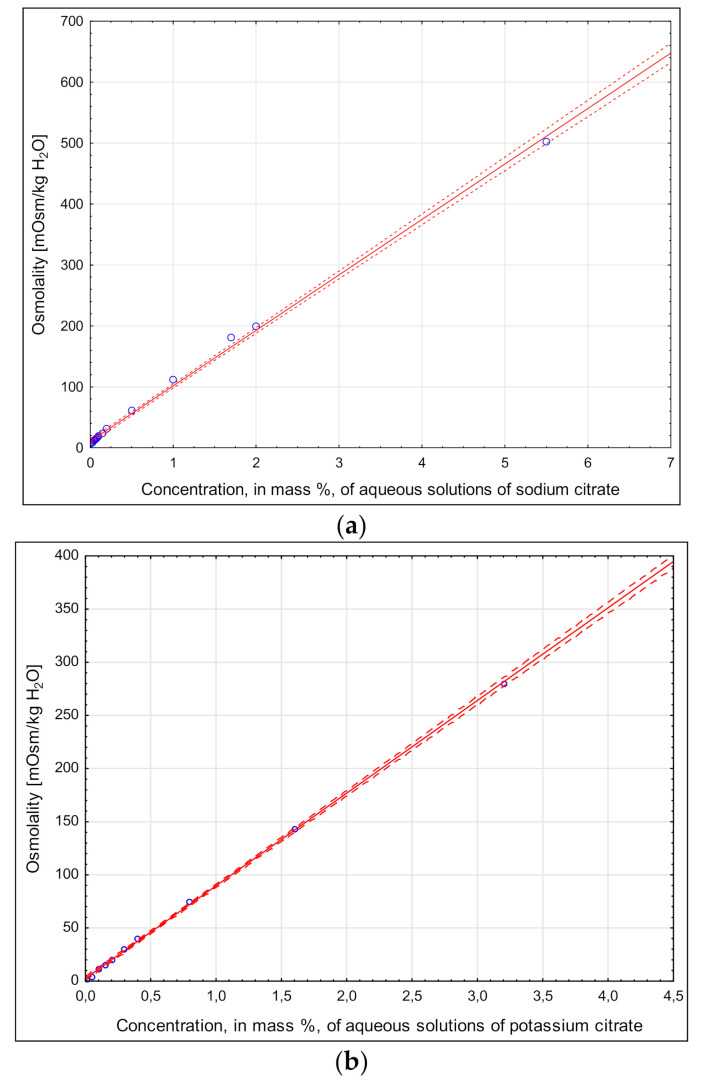

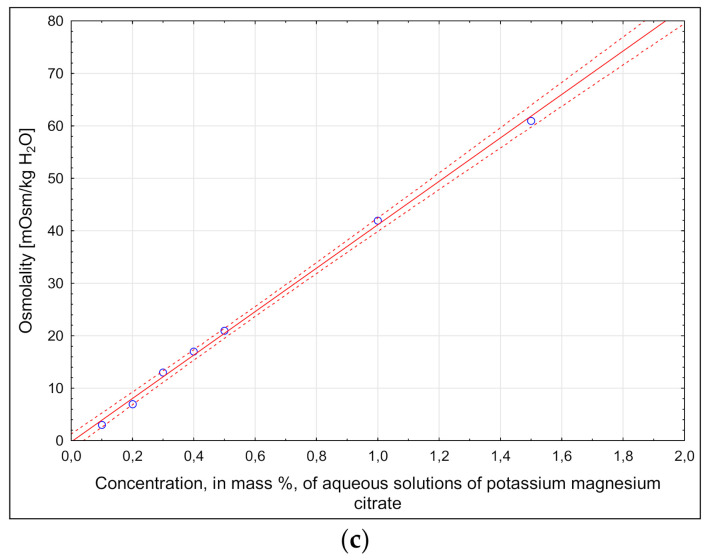
Electrolyte osmolalities in aqueous solution. (**a**) Sodium citrate: Osmolality = 10.58 + 91.04 × Concentration; r^2^ = 0.999.(**b**) Potassium citrate: Osmolality = 2.52 + 87.19 × Concentration; r^2^ = 0.999. (**c**) Magnesium citrate: Osmolality = −0.23 + 41.40 × Concentration; r^2^ = 0.999.

Figure 4a–c show the effects of increasing concentrations of the osmolalities from sodium citrate, potassium citrate and magnesium citrate. Osmolalities linearly increased between the tested parameters, with coefficients of variation being similar and amounting to 0.999. The obtained relationships were statistically significant (*p* < 0.05). Figure 4 demonstrates that the tested salts had low levels of osmolalities under the concentrations used, but nevertheless agreed with the recommended levels of sodium, potassium and magnesium in sports drinks [10]. In order to achieve the osmolality found in an aqueous solutions of isotonic drinks (approx. 260 mOsm/kg H_2_O), the concentration of sodium citrate or potassium citrate should be about 3%. The recommended maximum content in of these electrolytes in sport drinks is much lower: about 1100 mg/L for sodium (approx. equivalent to 4110 mg/L sodium citrate) and up to 225 mg/L for potassium (approx. equivalent to 589 mg/L potassium citrate) [10]. These values increase the osmolality of water drinks by approx. 45 mOsm/kg H_2_O for sodium citrate and approx. 6 mOsm/kg H_2_O for potassium citrate while maintaining both the taste and ensuring adequate hydration of the body of a physically active person [10]. A comparison of osmolalities of the tested salts sodium and potassium citrate (Figure 4) with the previously measured osmolalities of NaCl and KCl [22] showed about 2.5 times lower osmolality values, respectively. Similar relationships also occurred at the lower concentrations recommended for the designed functional drinks. This demonstrates the usefulness of these compounds in controlling osmolality in regeneration beverages, as it is difficult to achieve the appropriate osmolality recommended for regeneration beverages when a multi-component formulation is used.

### 2.2. The Composition and Osmolality of Designed Beverages

The relationships identified between osmolality and component concentrations were used to design six fruit juice drinks dedicated to regenerating the body following intense physical exercise (Table 1). Components included: electrolytes (Na^+^, K^+^, Mg^2+^), carbohydrates, raspberry powders (obtained via the three powdering methods: FBJD, FD and SD), protein and water-soluble fiber (inulin). The method of successive approximations was used to develop the recipe compositions of the beverages, and a series of tests were devised according to a base composition that contained water, 8–6% isomaltulose, 0.1% sodium citrate and 0.05% potassium citrate 0.05%; (the reference amounts given in the literature [10]).The amount of magnesium salt added corresponded to a magnesium requirement of approx. 30% RI [26]. Powders were added in amounts ranging from 3% to 8%, and osmolality measurements were taken from levels of powder added at to beverages that did not exceed the established maximum of 600 mOsm/kgH_2_O. It was assumed that the designed compositions would ensure adequate hydration of the body after intensive physical exercise, whilst delivering sufficient calories to regenerate the body. These calories were derived from carbohydrates contained within the powdered formulations along with the added isomaltulose. Isomaltulose was used in the designed beverages instead of the traditionally used sucrose or glucose, because it has a sweetness similar to these sugars, but also has valuable functional properties such as a low glycemic index of 32 (the glycemic index for glucose is 100, while for sucrose it is 60) [27]. Isomaltulose is a slowly digested carbohydrate that provides a steady and sustained supply of calories in the blood stream. Several studies have reported smaller increases (about 50% less) in blood sugar and insulin levels after consuming meals or drinks where sugar sources were replaced with isomaltulose. Moreover, isomaltulose ingestion has been associated with a greater ability to burn fat after meals and after exercise [25,28].

Osmolalities measured for individual beverage components indicated that the overall osmolality of the designed beverages depended mainly on the content of carbohydrates (i.e., the selected sugar and raspberry powders obtained by the various methods). Different amounts were therefore added, ranging from 4% to 8% for isomaltulose and 3% to 8% for fruit powders, depending on different osmolality and sensory considerations (i.e., sweet and sour taste, which had been determined by preliminary tests). The other remaining ingredients were added so that their levels were constant at 3%, and therefor appropriate from a nutritional perspective (i.e., containing selected protein and fiber (inulin)). The most advantageous functional features were found in the enzymatically hydrolyzed collagen proteins, which apart from their quite low osmolality also retained high stability in aqueous solution as well as at a low pH below the isoelectric point of proteins; this did not unduly restrict the amounts of fruit powders that could be added. Collagen preparations are currently widely used in dietary supplements intended for a wide demographic, and can prevent and treat osteoarticular diseases in athletes burdened by heavy physical exertion and cosmetic preparations. Collagen can also keep blood vessels, skin, hair and nails in a good condition [29]. Inulin is a water-soluble fiber with prebiotic properties that are well documented in the scientific literature. Furthermore, raspberry powders containing high levels of antioxidants such as polyphenols and vitamin C have been found to ameliorate the effects of oxidative stress caused by excessive physical exertion [23]. They are an important source of nutrients for the body’s needs, and can also act as a supplement. The designed drinks contained powders obtained using three different methods: the innovative FBJD and FD methods, which consisted of shredding whole raspberry fruits into 3–4% amounts, and the SD method, using raspberry juice at a 6–8% amount. This corresponds to an equivalent of about 20–30% content when taking fresh fruit or raspberry juice into consideration, respectively [30]. The spray-dried method yielded a 50% lower juice content because a 50% maltodextrin carrier was used for its preparation [31]. Such previously mentioned contents used in designer drinks were established through pilot testing, assuming that the total sugar and acid contents would provide appropriately acceptable levels of taste and osmolality. The actual taste of the drinks became degraded as greater amounts of powders were added beyond those shown in Table 1, and the flavor needed to be counteracted by the addition of more isomaltulose. In order to enhance rehydration rates for athletes and delay fatigue, functional beverages should be designed to contain 40–80 g/L of carbohydrates (at most 60–80 g/L) [10]. Nonetheless, carbohydrates at concentrations over 80 g/L in drinks may slow down gastric emptying and decrease the absorption of water from the intestines during exercise [10,16]. When added to the designed beverages, electrolytes, sodium citrate and potassium citrate resulted in concentrations of 0.1% (300 mg Na^+^/L) and 0.05% (200 mg K^+^/L), respectively, which is in keeping with those drink levels found in other studies [3,10,16]. Measured osmolalities in designed drinks are presented in Table 2 (ranging from 388 to 607 mOsm/kg H_2_O), together with those calculated from regression analyses made on individual drink components such as fruit powders, electrolytes, carbohydrates, protein and inulin. The drinks differed statistically significantly (*p* < 0.05) in terms of determined osmolality. Beverages with a higher amount of isomaltulose and fruit powders were characterized by significantly higher (*p* < 0.05) osmolalities than the other designed drinks (FBJD_1, FD_1 and SD_1 drinks were divided into the same homogeneous group) (Table 2). Beverages with smaller additions of isomaltulose and fruit powders (i.e., FBJD_2 and SD_2) were characterized by similar osmolalities (they belonged to the same homogeneous group), while the osmolality determined for the FD_2 drink was intermediate—it was significantly lower (*p* < 0.05) than that marked for the FBJD_1, FD_1 and SD_1 beverages, but significantly higher (*p* < 0.05) than that marked for FBJD_2 and SD_2 drinks (Table 2). The osmolalities shown in Table 2 reveal that the tested beverages were hypertonics, delivering electrolytes, carbohydrates, protein and fiber and rehydrating the body; thus, they would be particularly beneficial after any intensive exercise. Osmolalities were found to be lower in three of the tested beverages, ranging from 388 to 411 mOsm/kg H_2_O, whilst in the other three they were higher at 593–607 mOsm/kg H_2_O, falling within the lower boundaries of the 500–800 mOsm/kg H_2_O range considered as adequate for body rehydration. The osmolarity of sport drinks taken during exercise ranges from a minimum of 200 mOsm/L to a maximum of 330 mOsm/L [10]. The designed drinks should accordingly be classified as hypertonic sports drinks when drunk after any training. Table 2 shows the osmolality values of the tested drinks, which were calculated according to the sum total osmolality values derived from the regression equations (Figure 1, Figure 2, Figure 3 and Figure 4) for each component employed in the designed beverages, as well as the overall osmolality values. Table 2 shows that osmolalities were higher by 5–22% when calculating the beverage components via linear regression, which may have been due to interactions among the beverage components [16,22]. It can thereby be concluded that any assessment of beverage osmolality should be based on measuring the osmolality of each component used, as this enables the composition of beverages to be more rapidly optimized compared to the traditionally used method of successive approximations. Nevertheless, it should be assumed that experimental values will be higher by at least 5–8%, and that the developed, final optimized products will require additional, corrective osmolality measurements acquired through analysis. Comparing the results of the osmolality of the drinks designed in this study and the osmolality of beverages obtained in our previous research [22], similar differences between the calculated and measured values can be observed.

### 2.3. Physicochemical and Bioactive Properties of Designed Regenerative Drinks

Table 3 shows the contents of soluble solids and sugars, total polyphenol, vitamin C, pH and antioxidant properties in the recovery drinks designed using whole raspberry fruit powders (obtained via the FBJD and FD methods) and raspberry juice (achieved through the SD method). The obtained differences in all examined parameters were statistically significant (*p* < 0.05). The drinks were high in soluble solids containing mainly carbohydrates, acids, protein and soluble fiber inulin, but varied depending on the recipe (11.2–20.5 °Bx), with the sugar content ranging from 7% to 11%. Indeed, this is consistent with quantities reported for commercially available recovery drinks [20,24]. Research results presented in the literature [10,16,17,18,19] have recommended that carbohydrate levels of 60–80 g/L are needed in rehydrating sports drinks if they are to provide optimal support for performance and fatigue in both isotonic and slightly hypotonic drinks. These can be acquired in the form of sugars taken before, during and after training at levels of 4–6%, 6–9% and 9–10%, respectively.

The vitamin C content in the designed drinks ranged from 8 to 20 mg/100 g per beverage, so that drinking 300 g was enough to satisfy 30–75% of the body’s requirement [26]. Nutritionally significant amounts of polyphenol and antioxidant activities were found in the designed drinks and Sadowska et al. (2020) [23] has indeed reported that raspberry powders contain large amounts of polyphenols, vitamin C and antioxidant activity. Our designed fruit-based drinks differed from currently manufactured drinks in their contents of hydrolyzed collagen proteins, which are easily digestible in nutritionally significant amounts (approx. 9 g per a 300 g serving of the drink), the equivalent to daily recommended intakes in dietary supplements (up to 10 g per day) [29]. These drinks were also distinguishable in their contents of fiber (approx. 3 g per 100 g of drink) with prebiotic properties, which makes it possible to include the nutritional claim of these drinks being ‘a source of fiber’ [32].

### 2.4. Sensory Quality of the Designed Recovery Drinks

**Figure 5:**
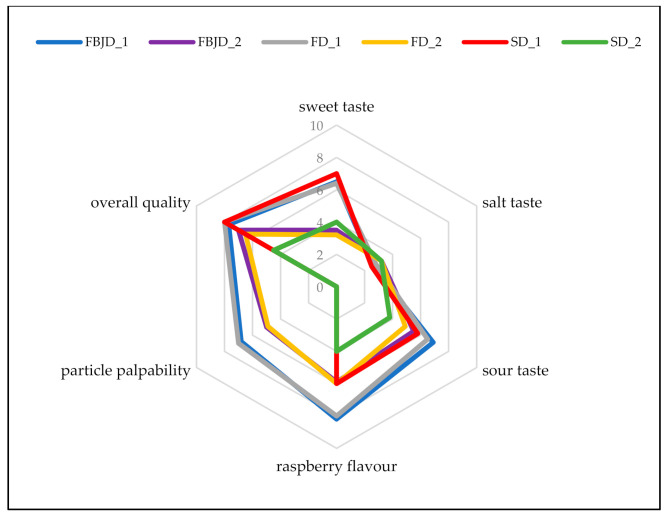
The sensory qualities of the designed recovery drinks.

Significant variations were seen in the sensory properties of the designed drinks (Figure 5). The finest tasting beverages (i.e., those with the highest palatability) had the highest additions of carbohydrates, due to their higher acidities resulting from the larger amounts of raspberry powder added, especially when prepared using the FBJD and FD powders. Overall quality was the highest for the FBJD 1, FD 1 and SD 1 versions of the designed drinks. Beverages with 8% or 6% isomaltulose were assessed differently for raspberry and sour taste due to larger quantities of whole fruit powders being added when obtained by FBJD and FD methods. Perceiving fruit particles obtained through spray drying has proven impossible in beverages. Our previous study [22] demonstrated the overall quality of fruit drinks to be the best when powdered blackcurrant fruit drinks were used that contained 4% freeze-dried powder and 5% sucrose with 6% spray-dried powders (including 60% maltodextrin and 3% glucose). Sucrose-containing drinks were rated much more highly than those with glucose, because the former has a more intensive sweetness on the human palate than glucose [33].

## 3. Materials and Methods

### 3.1. Recovery Drink Materials

Recovery drink materials consisted of raspberry powders obtained via the extraction three methods (i.e., the new fluidized-bed jet milling and drying (FBJD), freeze-drying (FD) and spray-dried (SD) methods), as well as other compounds such as isomaltulose (Tomex, Bielsko-Biała, Poland), prebiotic fiber (inulin) (Agnex, Białystok, Poland), protein preparations such as whey protein concentrate (80%) (Brenntag, Essen, Germany) and enzymatically hydrolyzed pork collagen (90%) (Intenson, Karczew, Poland). Minerals such as sodium, potassium and magnesium citrates (Chempur, Piekary Śląskie, Poland) were also used.

#### Raspberries Powder Preparation

Raspberry powders obtained via the FBJD method were obtained from a manufacturing plant in Radom (Poland). The production equipment comprised a grinding chamber device equipped with agitators with high-speed rotating blades that allowed the material to be dried and disintegrated after being introduced therein. This process was facilitated by an installed compressor that automatically controlled the air flow and temperature. A low-temperature drying was held at about 50 °C with an air flow velocity above 55 m/s. After being fed into the device, the raspberries were simultaneously dried and crushed by mutual collisions of particles in a high-energy air stream, which allowed the dried powder to be obtained. The obtained powders had a water content of approx. 2%. The detailed procedure for preparing FBJD powders is described in Sadowska et al. 2020 [23].

The FD raspberry powders were obtained by drying the raspberry fruits then turning them into a powder form. Freeze-drying of raspberry fruits, previously frozen at −30 °C, was performed by the Alpha Model 1–4 LSC freeze dryer (Martin Christ GmbH, Osterode am Harz, Germany) under the following conditions: time 48 h, pressure 10 Pa, drying chamber temperature −50 °C and shelf temperature 21 °C; these drying conditions were similar to those used by Si et al. (2016) [31]. The obtained dried fruits had a water content of approx. 1.6%. The post-FD dried raspberries were then ground to a powder form using a grinder with grinding knives (MKM 6003, Bosch, Stuttgart, Germany).

The spray-dried (SD) raspberry powders were prepared from raspberry juice with the addition of a 50% maltodextrin carrier (dextrose equivalent of DE 17) at air temperatures of 160 °C for inlets and 90 °C for outlets, where a 0.7 mm diameter dispersing nozzle was used (Buchi Mini Spray Dryer B—290, Flawil, Switzerland) [31]. The obtained powders had a water content of approx. 3%. After completing the drying processes, powders were packed into barrier packaging then stored ready for examination. Determinations were performed independently in triplicate.

### 3.2. Methods

#### 3.2.1. Osmolality

Osmolalities were determined using an OS-3000 Osmometer instrument (Trident med, Warsaw, Poland). Readings were expressed as mOsm/kg H_2_O. Osmolalities were measured for the following solutions: 2.5–15% raspberry powders, 1–16% isomaltulose, 1–10% inulin, 2.5–10% protein preparations, 0.17–5.5% sodium citrate, 0.2–6.5% potassium citrate and 0.5–3.3% magnesium citrate. Osmolality was also measured in each of the designed recovery drinks.

#### 3.2.2. The Soluble Solids Content

The soluble solid contents were measured in °Brix for each of the designed recovery drinks at 20 °C using a telescope refractometer (Eclipse Professional, Bellingham + Stanley Ltd., Saitama, Kent, Great Britain).

#### 3.2.3. The pH

The pH was measured in each of the designed recovery drinks using a laboratory-grade pH-meter (Elmetron, Zabrze, Polska).

#### 3.2.4. Assaying Directly Reducing Sugars and Total Sugars

This was determined by the Lane–Eynon method, according to an accredited Polish standard [34] and a manual of methods for the analysis of food [35]. Prior to assay, the beverages were first deproteinized using Carrez I and II reagents (Chempur, Piekary Śląskie, Poland). Sugars were hydrolyzed using concentrated hydrochloric acid (35–38%; Chempur, Piekary Śląskie, Poland).

#### 3.2.5. The Antioxidant Activity

Antioxidant activity was measured for each of the designed recovery drinks via the ABTS+•(2,2′-azino-bis-3-ethylben-zothiazoline-6-sulphonic acid) radical cation assay (Chempur, Piekary Śląskie, Poland) according to the modified method of Re et al. (1999) [36]. A predetermined fixed amount of the tested solutions, as previously established by a dilution scheme, was aliquoted into a 10 mL glass test tube, after which 3.0 mL of the radical cations ABTS+• was added to a PBS solution. Absorbance was measured after exactly 6 min of incubation at room temperature at 734 nm using a spectrophotometer (UV/Vis UV-6100A, Metash Instruments Co., Ltd., Shanghai, China). The results were represented through TEAC (Trolox Equivalent Antioxidant Capacity) as mmol Trolox in 100 g of each drink.

#### 3.2.6. The Total Content of Polyphenols

Total contents of polyphenols were measured in each of the designed recovery drinks by using the Folin–Ciocalteu (F–C) reagent (Chempur, Piekary Śląskie, Poland) according to the modified Singleton and Rossi (1965) method [37]. A predetermined fixed amount of the tested extract solution, as previously established by a dilution scheme, was aliquoted into a 50 mL flask, followed by adding 2.5 mL of the F–C reagent and 5.0 mL of 20% sodium carbonate (Chempur, Piekary Śląskie, Poland), with the remainder of the required volume composed of distilled water. Samples were incubated for 60 min at room temperature in darkness. The absorbance was measured at 720 nm using a spectrophotometer (UV/Vis UV-6100A, Metash Instruments Co., Ltd., Shanghai, China). Results were presented as mg GAE (Gallic Acid Equivalent) in 100 g of each drink.

#### 3.2.7. Vitamin C Assay

Vitamin C was measured in each of the designed recovery drinks as the sum of ascorbic acid and dehydroascorbic acid by means of HPLC with UV detection at a wavelength of 245 nm and a mobile phase flow rate of 0.8 mL/min. Total vitamin C sample content was determined after extraction for ascorbic acid and dehydroascorbic acid, followed by reduction with the dithiothreitol reagent (Sigma-Aldrich, Louis, MO, USA). HPLC separation was performed on a RP Symmetry C18.5 μm. 4.6 × 150 mm column at a temperature of 25 °C followed by detection using a UV2487 detector. The injection volume varied between 10 and 30 μL and results were expressed as the mg of vitamin C/100 g of each drink.

#### 3.2.8. The Sensory Analysis

The sensory characteristics of each designed recovery drink were determined using a scaling method established in ISO PN-EN ISO 13299:2016-05 (2016) [38] and carried out by an eight-person sensory panel of qualified assessors acknowledged as experts in all of the sensory methods (theoretical and practical) according to PN-EN ISO 8586:2014-03 [39]. Evaluations were carried out in two sessions. Six quality parameters were selected to evaluate the drinks: raspberry flavor, sour taste, sweet taste, salty taste, particle palpability and overall quality. The intensity of these were assessed on an unstructured 10-point scale in contractual units (cu.).

#### 3.2.9. The Statistical Analysis

Statistica 13.0 (Tibco Software Inc., Palo Alto, CA, USA) software was used for all statistical processing. The relationships between the concentration of individual beverage components in aqueous solutions and their osmolality were determined using the Pearson correlation coefficient. A one-way analysis of variance (ANOVA) was conducted with a post hoc analysis using the Duncan’s multiple range test, at a significance level *p* < 0.05, to investigate statistically significant differences between the evaluated parameters in the designed drinks.

## 4. Conclusions

The presented study has shown that it is feasible to make ready-to-drink, innovative fruit recovery drinks by dissolving fruit powders in 3–8% amounts (the equivalent of 20–30% fresh raspberries) following three different methods of powder preparation (i.e., FBJD, FD and SD). These powder-based drinks differed from those typically produced insomuch that the carbohydrate source was isomaltulose, which possesses a reduced glycemic index compared to glucose and sucrose, the ingredients most commonly used in recovery drinks. Further differences were the innovative use of fruit powders, the addition of easily digestible enzymatically hydrolyzed collagen protein and the inclusion of inulin, which possesses important prebiotic properties. Moreover, these designed drinks contained tailored amounts of electrolytes (Na^+^, K^+^ and Mg^2+^) that satisfied the recommended quantities of osmolalities, whilst concentrations of carbohydrates were consistent with the amounts found in the recovery drinks currently on the commercial market. The designed drinks also contained nutritionally valuable bioactive components such as vitamin C and polyphenols, derived from the raspberry powders (obtained by either the FBJD, FD or SD method). The powders prepared by the innovative FBJD method involved simultaneous drying and crushing of the fruit [24] and achieved the same level of quality as those obtained via the FD method; this level of quality was much higher than that obtained from SD powders. Sensory qualities of designed recovery drinks were satisfactory despite the matching levels of electrolytes observed in recovery beverages. The amounts of added fruit powders in the designed recovery drinks, however, were limited by sensory considerations (the taste was too sour). The concentrations of the drinks’ components in beverages were found to be linearly related to overall osmolality. Such outcomes may therefore help in designing effectively functional sports drinks, both in those without fruit components and in innovative fruit-based recovery drinks.

## Figures and Tables

**Table 1:** Recipe compositions of designed drinks.

Designed Drink Version		Raspberry Powder	Isomaltulose	Hydrolyzed Collagen	Inulin	Magnesium Citrate	Sodium Citrate	Potassium Citrate	Water
FBJD_1	g/100 g drink	4	8	3	3	1	0.1	0.05	81
	g/100 g powder	21	42	16	16	5	1	0.3	
FBJD_2	g/100 g drink	3	6	3		1	0.1	0.05	87
	g/100 g powder	23	46	23		8	1	0.4	
FD_1	g/100 g drink	4	8	3	3	1	0.1	0.05	81
	g/100 g powder	21	42	16	16	5	1	0.3	
FD_2	g/100 g drink	3	6	3		1	0.1	0.05	87
	g/100 g powder	23	46	23		8	1	0.4	
SD_1	g/100 g drink	8	6	3	3	1	0.1	0.05	79
	g/100 g powder	38	28	14	14	5	0.5	0.2	
SD_2	g/100 g drink	6	4	3		1	0.1	0.05	86
	g/100 g powder	42	28	21		7	1	0.4	

**Table 2:** Osmolalities of designed drinks.

Designed Drink Version	Osmolalities Calculated on the Basis of Regression Equations	Measured Osmolality [mOsm/kg H_2_O]	Difference [%]
FBJD_1	502	595.3 ± 5.0 ^c^	16
FBJD_2	367	398.3 ± 3.5 ^a^	8
FD_1	463	593.0 ± 6.0 ^c^	22
FD_2	331	411.7 ± 3.1 ^b^	19
SD_1	530	607.0 ± 5.0 ^c^	13
SD_2	367	388.0 ± 2.0 ^a^	5

Values are means ± standard deviation. ^a–c^—different letters in the same column are significantly different (Duncan’s test, *p* < 0.05). FBJD—fluidised-bed jet milling and drying; FD—freeze-drying; SD—spray-drying.

**Table 3:** Physicochemical and bioactive properties of the designed recovery drinks.

Designed Drinks Version	Soluble Solids [°Bx]	Sugars [g/100 g]	pH	Polyphenols [mg GAE/100 mL]	Antioxidant Properties [µM TEAC/100 mL]	Vitamin C [mg/100 mL]
FBJD_1	17.2 ± 0.3 ^c^	9.5 ± 0.6 ^c^	4.3 ± 0.01 ^a^	55.3 ± 0.9 ^d^	792.0 ± 17.5 ^d^	10.8 ± 0.1 ^b^
FBJD_2	11.2 ± 0.3 ^a^	6.9 ± 0.4 ^a^	4.4 ± 0.01 ^c^	44.0 ± 0.8 ^b^	730.9 ± 6.4 ^c^	8.1 ± 0.2 ^a^
FD_1	18.3 ± 0.6 ^d^	11.0 ± 0.2 ^e^	4.3 ± 0.01 ^a^	49.1 ± 0.7 ^c^	787.4 ± 6.0 ^d^	10.9 ± 0.4 ^b^
FD_2	13.3 ± 0.3 ^b^	7.3 ± 0.6 ^b^	4.3 ± 0.02 ^a^	42.0 ± 1.6 ^b^	715.2 ± 5.1 ^b^	8.2 ± 0.3 ^a^
SD_1	20.5 ± 0.5 ^e^	10.2 ± 0.7 ^d^	4.3 ± 0.03 ^a^	47.9 ± 1.6 ^c^	711.9 ± 1.9 ^b^	20.6 ± 0.0 ^d^
SD_2	13.3 ± 0.3 ^b^	7.9 ± 0.4 ^b^	4.3 ± 0.01 ^b^	27.2 ±1.3 ^a^	645.7 ± 2.7 ^a^	15.5 ± 0.1 ^c^

Values are means ± standard deviation. ^a–d^—different letters in the same column are significantly different (Duncan’s test, *p* < 0.05). FBJD—fluidised-bed jet milling and drying; FD—freeze-drying; SD—spray-drying.

## Data Availability

Not applicable.

## Sample Availability

Samples of the compounds are not available from the authors.
